# Exploration of the Relationships Among Narcissism, Life Satisfaction, and Loneliness of Instagram Users and the High- and Low-Level Features of Their Photographs

**DOI:** 10.3389/fpsyg.2021.707074

**Published:** 2021-08-26

**Authors:** Yunhwan Kim, Dongyan Nan, Jang Hyun Kim

**Affiliations:** ^1^College of General Education, Kookmin University, Seoul, South Korea; ^2^Department of Interaction Science, Sungkyunkwan University, Seoul, South Korea; ^3^Department of Human-Artificial Intelligence Interaction, Sungkyunkwan University, Seoul, South Korea

**Keywords:** Instagram, narcissism, life satisfaction, loneliness, low-level features, Microsoft Azure cognitive services

## Abstract

We examined the associations between the characteristics of Instagram users and the features of their photographs. Narcissism, life satisfaction, and loneliness were employed for user variables and the features at high- (content) and low-levels (pixel) were employed to analyze the Instagram photographs. An online survey was conducted with 179 university students, and their Instagram photographs, 25,394 in total, were collected and analyzed. High-level features were extracted using Computer Vision and Emotion Application Programming Interfaces (APIs) in Microsoft Azure Cognitive Services, and low-level features were extracted utilizing the program written by the authors. The results of correlation analysis indicate that narcissism, life satisfaction, and loneliness were significantly associated with a part of photograph features at high- and low-levels. The results of the predictive analysis suggest that narcissism, loneliness in total, and social loneliness could be predicted with acceptable accuracy from Instagram photograph features, while characteristics such as life satisfaction, family loneliness, and romantic loneliness could not be predicted. Implications of this research and suggestions for further research were presented.

## Introduction

Self-expression is one of the key behaviors concerning the use of social networking sites (SNSs). The users of SNSs can build and develop various relationships with other users; they can meet their offline friends again, have a conversation with others who share similar interests and ask questions about what they want to know. Despite the variety of relationships, the actual behaviors of users mainly consist of self-expression (Kim and Dindia, 2011; Taddei and Contena, 2013). The initialization of a relationship, asking for being friends (e.g., on Facebook) or following (e.g., on Instagram and Twitter), can be regarded as expressing the presence of and basic information about oneself (Barak and Gluck-Ofri, 2007). In addition, liking or commenting on the posts of other users can be considered as expressing their preference. The uploading of new posts, one of the most active online behaviors, can also be active self-expression (Orben and Dunbar, 2017) in that users can choose the topic of posts, content, and uploading time which reveals the explicitly and implicitly of the user.

In this regard, studies about social media posts have taken a large part of the literature on self-expression and social interaction on SNSs. Especially, much attention has been paid to the relationships between the characteristics of social media posts and the characteristics of their uploaders. Social media posts can be considered to reflect the characteristics of the user as they are both the instrument and the consequence of interaction. On one hand, users incorporate what they want to express in their posts, and other users like, comment on, and share them and thus, the posts are utilized as an instrument of interaction. On the other hand, posts are the consequence in that they are generated and remained throughout interaction among users as the footprint of their expressions and responses. Accordingly, analyzing social media posts, as well as conducting surveys to users, can be a good way to investigate the self-expression of the users of SNS (Sun and Wu, 2016).

In investigating the relationships between user characteristics and social media posts, it is a crucial issue to choose the characteristics of the user to be examined. While the Big-Five personality traits are the ones that have drawn the most attention (Vazire and Gosling, 2004; Marcus et al., 2006; Quercia et al., 2011; Winter et al., 2014; Ferwerda et al., 2016), narcissism, life satisfaction, and loneliness have also been the key characteristics of the user that have been explored in terms of their relationships with SNS content. According to previous research, the narcissism of SNS users is manifested in their content (Buffardi and Campbell, 2008) including photographs (Vazire and Gosling, 2004). Facebook content (Mehdizadeh, 2010), especially the topics in status updates of users (Marshall et al., 2015), is known to be related to the narcissistic trait of the users. Also, the life satisfaction of users is associated with the SNS use either positively (Cho et al., 2014), negatively (Sheldon and Bryant, 2016; Hawi and Samaha, 2017), or both (Chan, 2014). Furthermore, the life satisfaction of SNS users is expressed in content, such as smile intensity (Seder and Oishi, 2012), as well. Loneliness is related not only to the way users use SNS (Clayton et al., 2013; Yang, 2016) but also to the materials they post (Al-Saggaf and Nielsen, 2014); lonely SNS users posted more about their personal information than those who are not.

Although the relationships between characteristics of the user and SNS content have been much explored in many previous studies, several gaps still remain. First of all, past studies have analyzed mostly text rather than images or videos. While traits of SNS users can be revealed in photographs (Mehdizadeh, 2010) as much as in texts, not many prior studies analyzed SNS photograph data. Second, although there have been rare studies that tried to analyze SNS photograph data, the data analyzed were not the whole photographs of a given user but the limited kinds of photographs such as profiles (Gosling et al., 2011; Hum et al., 2011; Eftekhar et al., 2014) or selfies (McCain et al., 2016; Ridgway and Clayton, 2016; Sorokowska et al., 2016). Third, the main method adopted was content analysis by human coders (Döring et al., 2016; Simpson and Mazzeo, 2016; Tiggemann and Zaccardo, 2018); it made it difficult not only to examine a large number of photographs but also to adopt various low-level features which may convey other kinds of information that high-level features may not.

The present study aimed to explore the associations between characteristics of the Instagram user, narcissism, life satisfaction, and loneliness, and the features of photographs they uploaded. Exploring these, this study tries to fill the gaps that past studies had overlooked, as mentioned above. First, it analyses Instagram posts. Instagram is a photograph-centric SNS that has become popular especially among young generations. With the ease of use, many filters, and the advanced functionality of smartphone cameras, Instagram is one of the suitable platforms for online self-presentation, and it reflects the characteristics of the user through the photographs they posted. Next, this study analyses all photographs of a given user instead of certain kinds of photographs. Last, but not least, the uniqueness of this research mainly lies in the features that it utilizes in analyzing Instagram photographs. This study employs both high-level features which are related to the content of a photograph and low-level features which are related to the information in pixels of a photograph. In detail, the high-level features utilized are the content category, the number of faces on a photograph, and the emotion manifested in faces on a photograph. The low-level features utilized are the variance of RGB values and brightness in pixels in a photograph, and the share of pixels with warm colors in a photograph. There are mainly two tasks implemented with this research: (1) to examine the correlations between characteristics of the user and photograph features and (2) to identify whether the characteristics of the user would be predicted from Instagram photograph features. Therefore, the research questions of this research are as follows.

RQ1. How the narcissism, life satisfaction, and loneliness of Instagram users are related to the features of the photographs they upload?
RQ1-1. How are the characteristics of the user and photograph features correlated with each other?
RQ1-2. Can photograph features predict characteristics of the user in terms of narcissism, life satisfaction, and loneliness with a sufficient level of accuracy?

## Theoretical Framework

### Literature on Instagram

In the body of literature on Instagram, there have been studies that investigated why and how Instagram users use it: the motive of use and/or actual user behavior. Concerning the motive of use, Lee et al. (2015) reported that Instagram users had five primary motives: social interaction, archiving, self-expression, escapism, and peeking. In a similar vein, Sheldon and Bryant (2016) have shown that the main reasons for Instagram use were surveillance/knowledge about others, documentation, coolness, and creativity. They also found that characteristics of the users were related to the motive of Instagram use; for example, users who were good at social activity were more likely to use Instagram for documentation. Concerning use behavior, Araujo et al. (2014) revealed that Instagram users tended to spend their weekends and the night time watching posts and to endorse photographs that already had many likes and comments. Also, Ridgway and Clayton (2016) have shown that body image satisfaction positively affected the frequency of selfie-posting on Instagram. A few studies investigated the association between depression and Instagram use behavior; the work of Frison and Eggermont (2017) demonstrated that the type of Instagram use (browsing, posting, or liking) was related to the depressed mood. Those who used Instagram mainly for browsing have shown higher chances of having depressive moods than the others, and those who are in depression have higher chances of posting on Instagram. Lup et al. (2015) also reported that, for users who followed more strangers, frequent Instagram use was negatively related to depression but it was positively associated with users who followed fewer strangers. Belanche et al. (2020) found that regardless of the fit of new posts, Instagram users tended to continually interact with the accounts of the influencers. Casaló et al. (2020) demonstrated that perceived originality positively affects the behavioral intentions of Instagram users *via* the mediation of opinion leadership. Casaló et al. (2021) found that positive emotion played a notable role in inducing greater affective commitment of Instagram users to brand accounts. Casaló et al. (2017) reported that perceived originality positively affected intention of Instagram users by increasing perceived hedonism. Sanz-Blas et al. (2019) demonstrated that overuse of Instagram led to greater stress and emotional fatigue.

Meanwhile, there have been studies that analyzed Instagram photographs that met the certain condition for their contents in detail. Hosseinmardi et al. (2015) analyzed the Instagram photographs with cyberbullying-related hashtags. They found that, while photographs with certain kinds of content manifested association with being labeled as cyberbullying, many Instagram photographs containing profanity and cyber aggression were not labeled as cyberbullying. Santarossa et al. (2019) analyzed Instagram photographs tagged with #fitspo. Their results suggested that the largest content categories that the photographs belonged to were feeling good and appearance rather than sound, shape, or bad feelings, and that most #fitspo-tagged photographs manifested individuals with objectified poses. Tiggemann and Zaccardo (2018) focused on similar photographs tagged with #fitspiration; the content analysis showed that most photographs of women contained only thin and toned body types which might have negative effects on the body image of viewers. Guidry et al. (2015) conducted a content analysis of Instagram photographs with negative hashtags in the accounts of the 10 largest fast-food companies; they found that negative content was posted both by customers and employees and that it was due to the issues with service and the work environment. Filimonov et al. (2016) analyzed the content of Instagram photographs uploaded by Swedish parties during the 2014 elections campaign period and found that the parties utilized Instagram mainly for broadcasting campaign messages rather than mobilizing supporters.

A part of studies has focused on gender issues on Instagram. Geurin-Eagleman and Burch (2016) analyzed the Instagram photographs uploaded by men and women Olympic athlete and concluded that women athlete posted more about themselves and photographs were taken in private settings while male athletes posted photographs with more various topics. Duguay (2016) investigated how Instagram influenced the production and dissemination of selfies in terms of the visibility of lesbian, gay, bisexual, trans, and queer (LGBTQ) people based on actor-network theory using the range, reach, and salience. Other studies have paid attention to public health issues on Instagram. Sharma and De Choudhury (2015) extracted nutritional information from Instagram food photographs and investigated how users reacted to healthy and non-healthy food posts: people supported the pictures of healthy, moderate-caloric food significantly more than those of junk, high-caloric food. Holmberg et al. (2016) reported that adolescents post photographs of foods high in calories but low in nutrients on Instagram, and this result may be used to promote the health of the adolescent. Furthermore, Seltzer et al. (2015) explored how Instagram was used for public health information dissemination by analyzing photographs tagged with #ebola and reached the similar conclusion that Instagram can be used to assess public fears and to provide targeted interventions. Moreno et al. (2016) examined non-suicidal self-injury (NSSI) content on Instagram and found that they were often veiled by ambiguous hashtags and were not reliably detected by content advisory warnings.

As seen from the above, there have been many studies concerning various aspects of Instagram use. However, the content analysis method by human conders was mainly used in the literature and computational analysis of Instagram photographs has not been conducted sufficiently. While a small number of exceptional studies utilized computational methods for analyzing Instagram photographs (Ferwerda et al., 2016; Kim and Kim, 2018), the limited method has prevented researchers from both analyzing a large number of photographs and employing various features which require automated data processing. This study tries to fill this gap and conducts a computational analysis of Instagram photographs using features both at content and pixel levels.

### User Characteristics and the Features of Their Posts

In addition to the studies that have paid attention to motive of use of SNS users (Ross et al., 2009; Sheldon and Bryant, 2016), actual use behavior (Amichai-Hamburger and Vinitzky, 2010; Correa et al., 2010; Sorokowska et al., 2016), or both (Seidman, 2013), many studies have investigated how characteristics of SNS users are related to the features of the posts they upload. Among user characteristics, Big-Five personality traits have drawn the most attention in the literature (Vinciarelli and Mohammadi, 2014a,b). Among different formats of data, SNS posts in a text form have been examined mostly, while SNS photographs began to be taken into consideration recently. Regarding their relationship, it has been shown that SNS posts reveal the actual personality of the users, not self-idealization (Back et al., 2010) and that SNS users extend their offline personalities into an online domain (Gosling et al., 2011). Further, Youyou et al. (2015) suggested that computer-based personality judgments based on Facebook data can be more accurate than personality judgments of humans on others.

A group of studies has shown that the features extracted from the online text are not only associated with but also capable of predicting the personality of text uploaders. The work of Oberlander and Nowson (2006), which was one of the earliest of its kind indicated by Celli (2012), reported that personality traits of blog authors can be predicted using a corpus in their blog texts. In addition, some studies utilized the features extracted by the linguistic inquiry and word count (LIWC; Pennebaker et al., 2015) software to examine the online text and user personality. To name a few, Gill et al. (2009) demonstrated that the content of the blog was different significantly by the personality of bloggers. Golbeck et al. (2011) showed that the personality traits of Facebook users can be predicted by their profile text. Kern et al. (2014) analyzed Facebook posts and suggested that the use of particular words and phrases can provide insights into the personality traits of uploaders. Other studies further explored more sophisticated linguistic markers of text and their links with personality. Celli (2012) showed that 22 linguistic markers extracted from FriendFeed posts can predict the Big-Five personality traits of the uploaders. Qiu et al. (2012) conducted a similar study using Twitter data; their results suggested that Big-Five personality traits were manifested through specific linguistic markers in tweets. Schwartz et al. (2013) and Park et al. (2015) both took an open-vocabulary approach; instead of examining whether a specific set of words or phrases (closed-vocabulary) occurred in the target text, they took into account all words and phrases in the text and found their significant associations with Big-Five personality traits of text uploaders.

Other studies utilized SNS photographs for investigating their relationships with the characteristics of uploaders. Among many kinds of photographs, the most used was profile photographs; Eftekhar et al. (2014) employed 10 features concerning Facebook profile photographs (e.g., the total number of Facebook tagged photographs and the total number of uploaded photographs) for predicting Big-Five traits of the profile owners. Wu et al. (2015) categorized the content of Facebook profile photographs and showed that it reflected the personality of users. Twitter profile photographs were also found to be related to the personality of the account owner. The results in Liu et al. (2016) suggested the linkage between Big-Five personality traits and features of Twitter profile photographs. For example, more positive emotions were manifested in the profile photographs of more agreeable and conscientious users than those of the others.

However, many studies including the above did not analyze all photographs uploaded by a given user to examine the linkage to characteristics of the uploader. Their analyses were confined to limited kinds of photographs such as profiles or selfies. Except for few studies (Ferwerda et al., 2016; Skowron et al., 2016; Kim and Kim, 2018), research on user characteristics and features of posts was mainly conducted based on analysis of limited kinds of photographs on SNS. The present research tries to overcome this deficit and to make links among narcissism, life satisfaction, and loneliness of Instagram users and the photograph features extracted from all of their photographs.

### Narcissism, Life Satisfaction, Loneliness, and SNS Content

Social networking sites can be regarded as a suitable environment for narcissistic traits to be revealed for two reasons (Buffardi and Campbell, 2008). SNS consists of shallow relationships, and it is a highly controlled environment. On one hand, a large number of weak tie connections in SNS is an ideal outlet for narcissists to exhibit their self-loving traits (Bergman et al., 2011) because there is no need to logically prove their statement and there is no worry to be blamed for their ego-centric online behavior. On the other hand, narcissists can highly control the privacy of their posts and interaction, and thus, they can be dominant and competitive in an online social situation (Mehdizadeh, 2010). Thus, narcissists can be thought to post more self-promoting content on SNS than non-narcissists do (Buffardi and Campbell, 2008).

This relationship between the narcissism of SNS users and the characteristics of the content has been investigated in a large amount of literature. While some research suggested that the evidence for the relationship was weak (Skues et al., 2012; Paramboukis et al., 2016), results in a greater number of studies have shown that the narcissism of SNS users was manifested in the content they uploaded. Buffardi and Campbell (2008) reported that narcissism predicted frequent self-promoting posting on Facebook. The same conclusion has been drawn in many studies including Mehdizadeh (2010) and DeWall et al. (2011). Further, by analyzing the content of Facebook updates, Winter et al. (2014) suggested that a higher level of narcissism led to deeper self-disclosure as well as more self-promotional content. Wang et al. (2012) also reported that narcissistic SNS users updated their status more frequently and were more likely to upload photographs that might look more attractive. Similar results can be found in Moon et al. (2016) which stated that in comparison to non-narcissists, narcissists spent more time on Instagram, updated their profile more often, and posted more self-presented photographs, such as selfies.

A group of studies delved further. They subdivided narcissism and investigated their respective relationships with SNS content. For example, Carpenter (2012) made use of the sub-scales of narcissism, grandiose exhibitionism and entitlement/exploitativeness, and showed that the former can predict self-promoting behaviors (frequent status updates and selfie posting) on Facebook, while the latter failed to predict them. Also, McCain et al. (2016) subdivided narcissism into vulnerable and grandiose narcissism and indicated that the latter was associated with taking and posting more selfies, which is one of the self-promoting content. Barry et al. (2017) examined the relationships between some subcategories of narcissism and specific categories of selfies; they demonstrated that vulnerable narcissism significantly affected physical appearance selfies.

Other studies also delved further but in another way, specific kinds of SNS contents were investigated in terms of their relationship with the narcissism of their uploaders. Vazire et al. (2008) suggested that narcissistic traits can be manifested *via* physical signatures in photographs; narcissists were more likely to wear expensive and flashy clothing and they had an appearance that needs a lot of preparation. Ong et al. (2011) sub-divided the Facebook features into presenting self-generated (e.g., profile picture rating, status update frequency) and system-generated contents (e.g., social network size, photograph count) and suggested that narcissism of users predicted the former significantly but not the latter. Marshall et al. (2015) showed that narcissists used Facebook mainly for attention-seeking and this explained why they were more likely to update about their accomplishments such as diet and exercise. In addition, the selfie has been one of the most investigated contents in terms of its relationship with narcissism. While few studies suggested no relationship (Etgar and Amichai-Hamburger, 2017), more studies, including ones briefly mentioned above, found that selfie posting on SNS was related to narcissism (Fox and Rooney, 2015; Weiser, 2015; Kim et al., 2016). Especially, Sorokowski et al. (2015) subdivided their research sample by gender and showed that selfie posting was associated with narcissism among men but not among women.

Concerning life satisfaction, prior research on the influence of SNS use suggested contrasting results. A group of studies demonstrated that SNS use and life satisfaction were positively associated (Valenzuela et al., 2009; Oh et al., 2014). SNS use provided a greater benefit to users who have a low level of life satisfaction (Ellison et al., 2007) and, in turn, life satisfaction had an impact on the intention to use SNS (de Oliveira and Huertas, 2015). In contrast, other studies have shown that SNS use, especially excessive and addictive use, had a negative influence on life satisfaction (Błachnio et al., 2016; Hawi and Samaha, 2017) and, in turn, a higher level of life satisfaction led to a lower level of SNS use (Sheldon and Bryant, 2016). Chan (2014) suggested more mixed results, SNS use lowered extraversion and neuroticism of users both of which were related to life satisfaction.

Studies on different types of SNSs (e.g., Twitter, Facebook, Linked In) have demonstrated that the relationship between SNS use and life satisfaction can be reflected in the content users upload on SNSs. Pittman and Reich (2016) found that Instagram use had a positive relationship with life satisfaction, but Twitter use did not have such a linkage. Utz and Breuer (2019) implied that professional informational benefits positively affect LinkedIn use *via* the mediation of life satisfaction. Cho et al. (2014) reported that Facebook users who were more satisfied with their lives were more likely to be active in self-expression and their content tended to contain more about themselves. Going deeper, Seder and Oishi (2012) paid attention to the smile intensity in the photographs on Facebook. Based on previous studies which stated that the emotion expressed in photographs were associated with life satisfaction (Harker and Keltner, 2001; Freese et al., 2007; Hertenstein et al., 2009; Abel and Kruger, 2010), they showed that smile intensity manifested in Facebook photographs predicted the life satisfaction of their uploader. Also, Wang (2013) investigated whether life satisfaction was related to the use of location-based services on Facebook; one of their results suggested that the higher level of life satisfaction influenced the more frequency of check-in, the consequential content of location-based services.

Loneliness has been investigated in terms of its relationship with SNS. Previous research suggested that loneliness was one of the positive predictors of Facebook use (Clayton et al., 2013; Blachnio et al., 2016), although users were not emotionally attached to Facebook but only used it as a connection to others. Thus, the more the users felt lonely, the more they spent time on Facebook (Skues et al., 2012), which is why they had more Facebook friends. However, the lonely users did not actively participate in content creation but only passively consumed contents. Furthermore, a group of studies suggested that SNS use was different according to the sub-factors of loneliness. For example, Ryan and Xenos (2011) measured loneliness by three sub-factors, family, romantic, and social, as well as total loneliness utilizing the social and emotional loneliness scale for adults (SELSA-S; DiTommaso et al., 2004) and explored their relationships with Facebook use. It was one of their results that Facebook non-users had the higher level of loneliness; but it was the case only in social (friend) loneliness. Rather, Facebook users were higher in family loneliness. This indicates that loneliness needs to be explored both in total and in sub-factors when it comes to SNS use.

Social networking sites content has also been examined with regard to the loneliness that users perceive in their lives. Users who perceived more loneliness were found to upload more personal information (Al-Saggaf and Nielsen, 2014), and this self-disclosure reduced their level of loneliness (Deters and Mehl, 2013). Further, the use of photograph-centric SNS reduced the level of loneliness significantly more than the use of text-centric SNS (Pittman and Reich, 2016). Yang (2016) tried to associate the way of Instagram use and loneliness of users; it has been found that interaction and browsing were associated with a lower level of loneliness, whereas broadcasting was related to higher loneliness.

However, narcissism, life satisfaction, and loneliness have not been sufficiently investigated in terms of their relationships with social media photographs, especially with their low-level features. Given that narcissism, life satisfaction, and loneliness of SNS users are related to their usage behavior, the relationships are expected to be revealed in the photographs they uploaded. Further, pixel-level features, as well as content-level ones of their photographs, would reflect narcissism, life satisfaction, and loneliness of uploaders. This study tries to expand the scope of the literature by exploring the link among narcissism, life satisfaction, and loneliness of SNS users and the photograph features both at content and pixel levels.

The definitions of the variables used in this research are reported in Table 1.

**Table 1:** Definitions of life satisfaction, loneliness, and narcissism.

**Variables**	**Definitions**
Life satisfaction [Diener et al. (1985), Zhou et al. (2020), and Hong et al. (2021)	The overall assessments of people of the quality of their lives.
Loneliness [Yang (2016), Okruszek et al. (2020)	Unpleasant emotions caused by assessments of individuals of their social relationships.
Narcissism [Campbell et al. (2002), Buffardi and Campbell (2008), Moon et al. (2016)	A personality trait that has unrealistically positive self-views about status, appearance, social popularity, and intelligence of an individual.

## Methods

### Respondents and Procedure

An online questionnaire was conducted with 179 university students (mean age = 21.93 [*SD* = 2.39]; 70.4% women), who were Instagram users from Seoul and Gyeonggi area in South Korea. Women took a larger part than men in the sample, this could be a reflection of the general gender ratio in Instagram users of which about two-thirds are women (e.g., https://www.statista.com/statistics/530498/instagram-users-in-the-us-by-gender/). Participants responded to questionnaires about narcissism, life satisfaction, and loneliness. And, under their consents, all public photographs on their Instagram accounts were crawled applying InstaRipper (https://github.com/NitroXenon/instaripper). Participants were paid 10,000 KRW (~10 USD) for their participation. Non-active users who had uploaded <30 photographs were prohibited from participating and owners of accounts that seemed to be a fan account of public figures, such as rock stars, and accounts for commercial purposes were also prohibited.

### Materials

User variables and the photograph features at high and low levels were measured as described below. All user variables were measured by a 5-point Likert scale (Reuter et al., 2019; D'Adamo et al., 2020).

#### User Variables

A number of photographs. The number of Instagram photographs that a given user uploaded was counted.

Narcissism. Among 27 items of the short dark triad (SD3; Jones and Paulhus, 2014), which have been used in previous SNS research (McCain et al., 2016), nine items for narcissism were used.

Life satisfaction. The satisfaction with life scale (SWLS; Diener et al., 1985) was used to measure life satisfaction. It has been used in previous research on SNS including Facebook (Seder and Oishi, 2012).

Loneliness. The social and emotional loneliness scale for adults short form (SELSA-S; DiTommaso et al., 2004) was used to measure loneliness. Three sub-factors, family, social, romantic, of loneliness as well as total loneliness as their mean were measured.

The details of the questionnaires are reported in Appendix 1.

#### Photograph Features

There are high- and low-level photograph features operationalized in this study. The former is mainly about the advanced level of human perception and emotions including content categories and facial features, such as the number of faces and emotions revealed by facial expressions. The latter is in regards to the basic component of image files, such as pixels and brightness.

##### High-Level Photograph Features

Content category features. For a given photograph, it was determined which content category the photograph belongs to using computer vision API in Microsoft Azure cognitive services (https://azure.microsoft.com/services/cognitive-services/computer-vision/). For each uploaded photograph, it returns the category of the photograph among 86 sub-categories, and we recoded it into one of the top (upper) 15 categories; abstract, animal, building, dark, drink, food, indoor, others, outdoor, people, plant, object, sky, text, and transportation. Then, it was calculated how much share the photographs of each content category take among all photographs of a given user. For example, if an animal of a given user is 0.1, it means that 10% of all photographs that she uploaded were of animal-related content. Also, for a given content category distribution of users throughout 15 categories, the Gini coefficient (https://en.wikipedia.org/wiki/Gini_coefficient), which indicates the inequality of distribution, was calculated.

Face features. For a given photograph, features concerning the faces on the photograph were derived using emotion API in Microsoft Azure cognitive services (https://azure.microsoft.com/services/cognitive-services/emotion/). For each uploaded photograph, it detects human faces and determines the relative strength of eight emotions (anger, contempt, disgust, fear, happiness, neutral, sadness, and surprise) in each face so that the sum of all strengths becomes 1.

Based on these results, we first calculated the average number of human faces in all photographs of a given user. Also, the share of photographs with one human face (one-faced) among all photographs of a given user was calculated. We presumed that the photographs with one human face might be selfies. It was mainly for convenience; it was almost impossible to determine which face on photographs was of the owner of the account without any additional information. Yet, given that the participants of this research were university students, it was more likely that faces that appeared alone in a photograph were of themselves than that they were of their children as usual among in accounts of young moms. Further, since we excluded commercial and fan accounts from the sample, it was not probable that the faces were models of a product, rock stars, or political figures. Next, an average of eight emotions for each was calculated across all human faces detected all photographs of a given user. For example, if the happiness of a given user is 0.3, it means that the average relative strength of happiness on faces detected in all photographs of the user is 0.3.

##### Low-Level (Pixel) Photograph Features

The low-level (pixel) features of each photograph were calculated by the authors using Python programming language and OpenCV library.

Average means and variances of RGB pixel values. Each pixel in a given photograph has three numbers (0–255), red, green, and blue if represented by RGB format. We calculated the mean and variance of reds, greens, and blues, for each, in all pixels in a given photograph and took the averages of means and variances across all photographs of a given user. For example, red mean stands for the average of means of red values and red variance stands for the average of variances of red values in all pixels across all photographs of a given user. The variance exhibits the amount of contrast that influences the perceived color or brightness of the object (Brown and MacLeod, 1997), so the higher variance of brightness would make the photograph look brighter.

Average means and variances of saturations and values (i.e., brightness). Each pixel in a given photograph has three numbers (0–255, except hue), hue, saturation, and value (i.e., brightness) if represented by HSV format. For saturation and value, we calculated the mean and variance in all pixels in a given photograph, and took the averages of means and variances across all photographs of a given user. For example, saturation mean stands for the average of means of saturation values and saturation variance stands for the average of variances of saturation values in all pixels across all photographs of a given user.

Hue in a pixel stands for the kind of its color. We divided the total range of hue (0–179 in OpenCV) into intervals ([7, 23, 35, 90, 130, and 169]) for each color (orange, yellow, green, blue, violet, and red) and calculated the share of pixels for each color in a given photograph. Further, we calculated the share of pixels with warm colors (red, orange, and yellow) in a given photograph. In addition, the shares were averaged across all photographs of a given user. For instance, orange share means the average of shares of the pixel with orange color across all photographs of a given user.

## Results

A correlation analysis was conducted between user variables and photograph features. The full correlation table is presented in Appendix 2. The result shows that narcissism of Instagram users was correlated with a part of high-level features of photographs they uploaded (as shown in Table 2); photographs of narcissistic users were more likely to be people-related, contained more human faces, and expressed happier emotion on faces on average. In addition, photographs with one human face took more share in the photographs of narcissistic users. In contrast, the number of uploaded photographs and low-level features of Instagram photographs did not show a significant correlation with narcissism of uploaders. It indicates that narcissistic users in the sample did not upload more photographs than non-narcissistic users and their photographs did not show a significant difference at pixel level with the photographs of non-narcissistic users.

**Table 2:** Correlations between narcissism and a part of high- and low-level features of Instagram photographs.

	**N. of Photos**	**People**	**Avg. N. of Faces**	**One-Faced**	**Happiness**	**RGB Var**	**Value Var**	**Warm Share**
Narcissism	0.085	0.204^*^	0.175^*^	0.247^*^	0.172^*^	R 0.137G 0.085B −0.009	0.128	0.132

* *p < 0.05*.

As seen in Table 3, life satisfaction was positively correlated with the average number of human faces per photograph and the average happiness expressed on human faces on photographs. In other words, the photographs of users who were more satisfied with their lives contained more human faces and expressed happier emotion on faces. A significant correlation was not observed in all low-level features.

**Table 3:** Correlations between life satisfaction and a part of high- and low-level features of Instagram photographs.

	**N. of Photos**	**People**	**Avg. N. of Faces**	**One-Faced**	**Happiness**	**RGB Var**	**Value Var**	**Warm Share**
Life satisfaction	0.130	0.102	0.175^*^	0.077	0.185^*^	R 0.075G 0.055B 0.094	0.067	0.104

* *p < 0.05*.

Since we measured loneliness both in total and in three sub-factors, correlations with photograph features were analyzed in both aspects (as shown in Table 4). Loneliness in total was negatively correlated with a part of high-level features of Instagram photographs: photographs of lonely users contained a smaller number of human faces, expressed less happy emotion on faces, and were more animal-related. In addition, loneliness showed significant negative correlations with many low-level features of Instagram photographs as photographs of lonely users were less diverse in red, green, blue color and they were less diverse in brightness as well. In addition, pixels with warm colors took less share in their photographs.

**Table 4:** Correlations between loneliness and part of high- and low-level features of Instagram photographs.

	**N. of Photos**	**People**	**Animal**	**Avg. N. of Faces**	**Happiness**	**RGB Var**	**Value Var**	**Warm Share**
Loneliness	−0.051	−0.122	0.177^*^	−0.181^*^	−0.192^*^	R −0.228^*^G −0.226^*^B -−0.219^*^	−0.209^*^	−0.222^*^
Family loneliness	−0.071	−0.180^*^	0.099	−0.218^*^	−0.228^*^	R −0.155^*^G −0.157^*^B −0.161^*^	−0.132	−0.129
Social loneliness	−0.007	−0.052	0.006	−0.140	−0.114	R −0.105G −0.044B −0.071	−0.100	−0.115
Romantic loneliness	−0.027	−0.041	0.193^*^	−0.064	−0.086	R −0.188^*^G −0.212^*^B −0.187^*^	−0.178^*^	−0.192^*^

* *p < 0.05*.

Going deeper into sub-factors, it was mainly due to family loneliness that total loneliness was correlated with the average number of human faces per photograph and the average happiness expressed on human faces on photographs. Furthermore, it was mainly due to romantic loneliness that total loneliness was correlated with the share of animal-related photographs. Regarding low-level features of Instagram photographs, family loneliness and romantic loneliness showed negative correlations with the average variances of RGB pixel values, and romantic loneliness showed negative correlations with the average variance of brightness and the average share of pixels with warm colors.

In addition to correlation analysis, predictive models were built to investigate how accurately narcissism, life satisfaction, and loneliness could be predicted from the Instagram photograph features. Linear regression and random forest regression models were trained with 10-fold cross-validation and their root mean square errors (RMSEs) are presented in Table 5. In previous studies that predicted the characteristics of social media user from the feature of their posts where the same five-point scale was used, the RMSEs lied between 0.69 and 0.88 (Quercia et al., 2011) and between 0.66 and 1.01 (Ferwerda et al., 2016). Having these in mind, the results in Table 5 suggest that narcissism, loneliness in total, and social loneliness could be predicted with acceptable accuracy while the predictive power of Instagram photograph features over life satisfaction, family loneliness, and romantic loneliness were unsatisfactory.

**Table 5:** Root mean square error in 10-fold cross-validation.

**Target Variable**	**Linear Regression**	**Random Forest**
Narcissism	0.637	0.646
Life satisfaction	0.956	0.834
Loneliness	0.654	0.625
Family loneliness	0.892	0.894
Social loneliness	0.627	0.629
Romantic loneliness	1.378	1.359

## Discussion

In the present study, we investigated the relationships between the characteristics of Instagram users, narcissism, life satisfaction, and loneliness, and the features of their photographs at high- and low-levels. We tried to associate user variables, measured by the traditional survey method, with photograph features, measured by a novel computational method, and draw conclusions about which users upload which photographs. The main findings of them are as follows.

First of all, the results of this study suggested that the content, not the number, of Instagram photographs is one of the key indicators of narcissism of the users. The level of narcissism was not associated with the number of Instagram photographs uploaded. This means that narcissists in our sample did not self-promote *via* uploading many photographs on Instagram. In spite of the general belief that SNS users would have a higher level of narcissism (Ryan and Xenos, 2011; Brailovskaia and Bierhoff, 2016) and would upload more photographs to self-promote, the result of this research indicates that they did not. Instead, the level of narcissism was associated with the high-level photograph features that can be interpreted as being self-promoting; more people-related content, more faces on photographs, and more happiness on faces. This finding corresponds with the previous research (Bergman et al., 2011) that demonstrated narcissism was not related to the amount of time or frequency of SNS use but the reason why users use it. Narcissists did not spend much time or update status frequently, but they wanted to have many SNS friends, wanted friends to know and to be interested in what they are doing, and made their profile show a positive image (Bergman et al., 2011). Further, this can be linked to the results in previous research that narcissists uploaded more self-promoting materials on SNS (Buffardi and Campbell, 2008; Mehdizadeh, 2010; Marshall et al., 2015). In those studies, narcissists wanted themselves to be shown attractive, social, and happy. Since narcissistic trait was related to the size of online social network (Bergman et al., 2011; Carpenter, 2012), one can hypothesize that the Instagram photographs that narcissists uploaded would contain more people-related content, and the result of this research can affirm the hypothesis. It has been also found that the faces on Instagram photographs of narcissists manifested more happiness. These corroborate the above statement that the content of Instagram photographs indicates narcissism of users.

Second, among many aspects of photograph content, selfies reflected narcissism of the users. Selfie is one of the representative phenomena that narcissism influences the way individuals use technology (Ong et al., 2011), and the result of this research indicates that selfies took more share in Instagram photographs of the users with a higher level of narcissism. This result corresponds with many previous studies which suggested a significant relationship between narcissism and selfie uploading (Carpenter, 2012; McCain et al., 2016), and it is also consistent with the above discussion that narcissists wanted to show their SNS friends what they are doing.

Third, the life satisfaction of Instagram users demonstrated complex relationships with the features of their photographs. While previous research suggested that SNS users who were more satisfied with their lives were more active in self-disclosure on SNS (Cho et al., 2014), the results in this study indicate that Instagram users with a higher level of life satisfaction did not upload more photographs than other users. In addition, while life satisfaction was known to be associated with positive relationships with others (Diener and Seligman, 2002), this was not manifested *via* a larger share of photographs with people-related content in our sample. It needs to be noted, however, that users with a higher level of life satisfaction uploaded photographs that contained many human faces. This tells us that they did not upload people-related photographs a lot, but once they did, the photographs expressed rich interactions with others which were expressed through many faces in their photographs. And it also needs to be noted that life satisfaction was not correlated with the share of selfies but was with the happiness expressed on faces on photographs. We can infer, from these results, that the happiness expressed on the faces in the photographs of users with higher life satisfaction was not only of the users themselves (as usually was in selfies) but also of people that they interacted with.

Fourth, Instagram users with a higher level of loneliness did not upload more photographs. This result might be inconsistent with previous research that suggested the level of loneliness was associated with the amount of SNS use (Clayton et al., 2013; Deters and Mehl, 2013; Blachnio et al., 2016). In addition, the result in another study that photograph-based SNS can reduce loneliness more than text-based one (Pittman and Reich, 2016) may look contrasting with the result of the present study. This inconsistency can be resolved if we take the SNS using behavior into consideration; users with a higher level of loneliness might use SNS a lot, especially photograph-centric SNS that can lessen their loneliness more, but it does not necessarily mean they upload more photographs. Ryan and Xenos (2011) suggested lurking behavior on SNS: lonely SNS users spend much time on SNS but they do not actively participate but use only passive functions or just look at posts and comments of others. This means that lonely users only lurk on SNS and the result of this research suggests that it is also the case with Instagram.

Fifth, as was narcissism above, loneliness was manifested in the content, not the number, of Instagram photographs. There were fewer numbers of human faces on the photographs of lonely users and the faces expressed less happiness. These results suggest that lonely Instagram users did not pretend to be not lonely and reveal their loneliness frankly to some extent. Further, this disclosure was also distinct at low-level features of their photographs; in contrast to narcissism and life satisfaction, loneliness showed a more remarkable (negative) correlation with all of the low-level features that might make their photographs brilliant and gorgeous (high variance in color and brightness and larger share in warm colors). Since, as mentioned above, lonely users might use SNS mainly in a passive way (Ryan and Xenos, 2011), they did not need to upload brilliant and gorgeous photographs. This result indicates that loneliness can be well-detected from the low-level features of Instagram photographs.

Finally, each sub-factor of loneliness explained the correlation of total loneliness with each photograph feature. Family loneliness was the key factor of loneliness that was associated with the share of people-related photographs, number of human faces, and happiness on faces. It needs to be noted that the share of photographs with animal-related content was significantly associated with romantic loneliness. It is well-known that animal decreases loneliness (Stewart et al., 2014), but its relationship with romantic loneliness, in particular, is under-researched and would be a further research topic. Also, romantic loneliness was the key factor of loneliness that made the photographs less brilliant and less gorgeous; RGB colors and brightness were less diverse, and warm colors took a smaller share.

The main limitation of this research is its limited scope of samples. That is, 179 university students responded to the survey in this study, which represents a part of the young generation. Further research should validate the results of this research by analyzing a larger sample size or diverse age groups. In particular, future research could be conducted for cross-cultural comparison: how the correlations between user variables and photograph features would be different in other cultures. In addition, more low-level photograph features could be employed to test their correlation with user variables. For example, fractal dimension (Kim et al., 2014), texture, and harmony (Machajdik and Hanbury, 2010) are the low-level features that have been used in previous studies.

Overall, the present research may contribute to the body of research on online self-presentation. It presented the way how user characteristics were manifested in Instagram photographs. It can be said that its uniqueness lies in the link between user variables and photograph features both at high- and low-levels. The results demonstrated that photograph features at low-level, as well as high-level features, can be indicators of the characteristics of users.

## Data Availability Statement

The original contributions presented in the study are included in the article/Supplementary Material, further inquiries can be directed to the corresponding author/s.

## Ethics Statement

Ethical approval was not provided for this study on human participants because it is not necessary. The patients/participants provided their written informed consent to participate in this study.

## Author Contributions

YK: research design, implementation, and writing. DN: writing and editing. JK: writing, editing, and research design. All authors contributed to the article and approved the submitted version.

## Conflict of Interest

The authors declare that the research was conducted in the absence of any commercial or financial relationships that could be construed as a potential conflict of interest.

## Publisher's Note

All claims expressed in this article are solely those of the authors and do not necessarily represent those of their affiliated organizations, or those of the publisher, the editors and the reviewers. Any product that may be evaluated in this article, or claim that may be made by its manufacturer, is not guaranteed or endorsed by the publisher.
