# Supplementary material for: Exploration of the Relationships Among Narcissism, Life Satisfaction, and Loneliness of Instagram Users and the High- and Low-Level Features of Their Photographs
Source: Front Psychol. 2021 Aug 26;12:707074. doi: 10.3389/fpsyg.2021.707074 (PMC8427304; doi:10.3389/fpsyg.2021.707074)
Supplement: Supplementary file 1 [file Data_Sheet_1.docx]

**Appendix 1. Questionnaire items**

| Factors | Items |
| --- | --- |
| Life satisfaction (Diener et al., 1985) | 1. In most ways my life is close to my ideal. |
|  | 2. The conditions of my life are excellent. |
|  | 3. I am satisfied with my life.  4. So far I have gotten the important things I want in life.  5. If I could live my life over, I would change almost nothing. |
| Narcissism (Jones & Paulhus, 2014) | 1. People see me as a natural leader. |
|  | 2. I hate being the center of attention. (reversal) |
|  | 3. Many group activities tend to be dull without me.  4. I know that I am special because everyone keeps telling me so.  5. I like to get acquainted with important people.  6. I feel embarrassed if someone compliments me. (reversal)  7. I have been compared to famous people.  8. I am an average person. (reversal)  9. I insist on getting the respect I deserve. |
| Social loneliness (DiTommaso et al., 2004) | 1. I feel part of a group of friends. |
|  | 2. My friends understand my motives and reasoning. |
|  | 3. I don’t have any friends who share my views, but I wish I did.  4. I am able to depend on my friends for help.  5. I do not have any friends who understand me, but I wish I did. |
| Family loneliness (DiTommaso et al., 2004) | 1. I feel alone when I am with my family.  2. There is no one in my family I can depend on for support and  encouragement, but I wish there was.  3. I feel close to my family.  4. I feel part of my family.  5. My family really cares about me. |
| Romantic loneliness (DiTommaso et al., 2004) | 1. I have a romantic partner with whom I share my most intimate  thoughts and feelings. |
|  | 2. I have a romantic or marital partner who gives me the support and encouragement I need |
|  | 3. I wish I had a more satisfying romantic relationship.  4. I have a romantic partner to whose happiness I contribute.  5. I have an unmet need for a close romantic relationship. |
