# Supplementary material for: Exploration of the Relationships Among Narcissism, Life Satisfaction, and Loneliness of Instagram Users and the High- and Low-Level Features of Their Photographs
Source: Front Psychol. 2021 Aug 26;12:707074. doi: 10.3389/fpsyg.2021.707074 (PMC8427304; doi:10.3389/fpsyg.2021.707074)
Supplement: Supplementary file 2 [file Data_Sheet_2.docx]

**Appendix 2. Correlation table**

|  |  | narcissism | life satisfaction | loneliness | family loneliness | social loneliness | romantic loneliness |
| --- | --- | --- | --- | --- | --- | --- | --- |
|  | N. of photos | .085 | .130 | -.051 | -.071 | -.007 | -.027 |
| content  category  features | abstract | -.038 | -.062 | .128 | .164* | .080 | .048 |
|  | animal | -.197* | .018 | .177* | .099 | .006 | .193* |
|  | building | -.078 | -.095 | .019 | -.014 | -.015 | .044 |
|  | dark | .000 | -.023 | .156* | .082 | -.050 | -.198* |
|  | drink | .037 | .027 | .044 | -.026 | .041 | .063 |
|  | food | -.070 | -.018 | -.073 | -.063 | .019 | -.075 |
|  | indoor | .085 | .023 | -.109 | .018 | -.081 | -.134 |
|  | others | -.114 | .037 | .142 | .198* | .114 | .032 |
|  | outdoor | .027 | -.007 | -.044 | -.040 | -.106 | .008 |
|  | people | .204* | .102 | -.122 | -.180* | -.052 | -.041 |
|  | plant | -.071 | -.099 | .049 | .104 | -.088 | .044 |
|  | object | -.044 | -.115 | .118 | .139 | .149* | .018 |
|  | sky | -.049 | -.066 | .064 | .068 | -.019 | .059 |
|  | text | -.090 | -.076 | .100 | .148* | .096 | .011 |
|  | transportation | -.084 | -.066 | .114 | -.042 | .178* | .114 |
|  | Gini | .040 | .086 | -.039 | -.099 | .044 | -.014 |
| face  features | avg. N. faces | .175* | .175* | -.181* | -.218* | -.140 | -.064 |
|  | one-faced | .247* | .077 | -.100 | -.127 | -.054 | -.042 |
|  | anger | .193* | .020 | -.079 | -.092 | .037 | -.074 |
|  | contempt | .148* | .133 | -.179* | -.102 | -.137 | -.137 |
|  | disgust | .123 | .151* | -.116 | -.104 | -.095 | -.062 |
|  | fear | .117 | .100 | -.036 | -.105 | .025 | .003 |
|  | happiness | .172* | .185* | -.192* | -.228* | -.114 | -.086 |
|  | neutral | .211* | .030 | -.053 | -.076 | -.036 | -.014 |
|  | sadness | .145 | .184* | -.122 | -.147 | -.125 | -.030 |
|  | surprise | .039 | .030 | .022 | -.002 | .156* | -.036 |
| pixel  features | blue mean | -.064 | .051 | -.014 | -.037 | .049 | -.019 |
|  | blue var | -.009 | .094 | -.219* | -.161* | -.071 | -.187* |
|  | green mean | -.072 | .068 | -.055 | -.055 | .041 | -.064 |
|  | green var | .085 | .055 | -.226* | -.157* | -.044 | -.212* |
|  | red mean | -.056 | .101 | -.110 | -.085 | .026 | -.118 |
|  | red var | .137 | .075 | -.228* | -.155* | -.105 | -.188* |
|  | sat. mean | -.004 | .017 | -.090 | -.017 | -.055 | -.096 |
|  | sat. var | -.049 | .031 | -.119 | -.051 | -.022 | -.132 |
|  | value mean | -.068 | .088 | -.089 | -.071 | .034 | -.100 |
|  | value var | .128 | .067 | -.209* | -.132 | -.100 | -.178* |
|  | red share | .123 | .139 | -.161* | -.127 | -.060 | -.128 |
|  | orange share | .075 | .057 | -.144 | -.093 | -.111 | -.102 |
|  | yellow share | -.035 | -.103 | .022 | .108 | .077 | -.069 |
|  | green share | -.201* | -.112 | .234* | .202* | .186* | .131 |
|  | blue share | -.054 | -.067 | .147* | .050 | .041 | .165* |
|  | violet share | - | - | - | - | - | - |
|  | warm share | .132 | .104 | -.222* | -.129 | -.115 | -.192* |

(* : p < .05, sat. : saturation)
